# Prevalence of multiple chronic conditions in the United States' Medicare population

**DOI:** 10.1186/1477-7525-7-82

**Published:** 2009-09-08

**Authors:** Kathleen M Schneider, Brian E O'Donnell, Debbie Dean

**Affiliations:** 1 Buccaneer Computer Systems and Service Inc., 1401 50thStreet, Suite 200, West Des Moines, Iowa 50266, USA

## Abstract

In 2006, the Centers for Medicare & Medicaid Services, which administers the Medicare program in the United States, launched the Chronic Condition Data Warehouse (CCW). The CCW contains all Medicare fee-for-service (FFS) institutional and non-institutional claims, nursing home and home health assessment data, and enrollment/eligibility information from January 1, 1999 forward for a random 5% sample of Medicare beneficiaries (and 100% of the Medicare population from 2000 forward). Twenty-one predefined chronic condition indicator variables are coded within the CCW, to facilitate research on chronic conditions.

The current article describes this new data source, and the authors demonstrate the utility of the CCW in describing the extent of chronic disease among Medicare beneficiaries. Medicare claims were analyzed to determine the prevalence, utilization, and Medicare program costs for some common and high cost chronic conditions in the Medicare FFS population in 2005. Chronic conditions explored include diabetes, chronic obstructive pulmonary disease (COPD), heart failure, cancer, chronic kidney disease (CKD), and depression.

Fifty percent of Medicare FFS beneficiaries were receiving care for one or more of these chronic conditions. The highest prevalence is observed for diabetes, with nearly one-fourth of the Medicare FFS study cohort receiving treatment for this condition (24.3 percent). The annual number of inpatient days during 2005 is highest for CKD (9.51 days) and COPD (8.18 days). As the number of chronic conditions increases, the average per beneficiary Medicare payment amount increases dramatically. The annual Medicare payment amounts for a beneficiary with only one of the chronic conditions is $7,172. For those with two conditions, payment jumps to $14,931, and for those with three or more conditions, the annual Medicare payments per beneficiary is $32,498.

The CCW data files have tremendous value for health services research. The longitudinal data and beneficiary linkage within the CCW are features of this data source which make it ideal for further studies regarding disease prevalence and progression over time. As additional years of administrative data are accumulated in the CCW, the expanded history of beneficiary services increases the value of this already rich data source.

## Background

The presence of chronic conditions has become epidemic. In the United States over 133 million people, or nearly half of the population, suffer from a chronic condition [1]. The high prevalence of chronic disease among the Medicare population has been well documented [2,1]. Of particular concern is the fact that many people suffer from not one, but multiple chronic conditions [3].

A new data source from the Office of Research, Development, and Information at the Centers for Medicare & Medicaid Services (CMS) was used for this study. Section 723 of the Medicare Modernization Act of 2003 (MMA) mandated a plan to improve the quality of care and reduce the cost of care for chronically ill Medicare beneficiaries. An essential component of this plan was to establish a research database that contained Medicare data, linked by beneficiary, across the continuum of care. CMS contracted with Buccaneer Computer Systems and Service Inc. (BCSSI) to establish the Chronic Condition Data Warehouse (CCW). Researchers interested in obtaining CCW data files should contact the CMS Research Data Assistance Center (ResDAC) [4]. The CCW was designed to facilitate chronic disease studies of the Medicare population. The database was made available to researchers in 2006 and has been used to provide data to many chronic disease researchers to date. Due to the newness of the database, this is believed to be one of the first publications of chronic disease statistics using CCW data. More information regarding the CCW can be found at [5].

Twenty one condition indicators are available from the Chronic Condition Data Warehouse (CCW). These predefined conditions include a combination of common and chronic conditions among older adults, and were designed to allow for streamlined data extraction of disease cohorts from the CCW. The 21 condition variables specify whether each Medicare beneficiary received services during the time frame to indicate treatment for these conditions; that is, the chronic condition variables indicate the clinical "presence" of the conditions as inferred from the pattern of diagnosis and procedure codes appearing in the fee-for-service (FFS) claims data. Six high frequency and high cost chronic conditions were selected for study (note: four types of cancer were combined into one "cancer" variable, in order to limit the count of conditions for these analyses). The six conditions are of particular interest in this paper because: 1) they are highly prevalent conditions in older adults, 2) they are commonly targeted in disease management programs in the U.S. [6], and 3) "presence" indicators were available in CCW datasets and could easily be used to define the cohorts. The conditions examined include cancer, chronic kidney disease (CKD), chronic obstructive pulmonary disease (COPD), depression, diabetes, and heart failure (HF). Current data support the high prevalence of these conditions [3,7].

A high proportion of older adults suffer from cancer, and an estimated 1 in 15 women 70 years or older will be diagnosed with breast cancer [8]. One in six men will be diagnosed with prostate cancer - with a median age for diagnosis at 68 years [9]. Cancer is the leading cause of death among people 60-79 years of age. In 2006 it was estimated that COPD affected approximately 7 million adults 65 years or older [10]. Hospitalizations for HF increase with age. Among the population aged 65-84 years old, there were 18.8 hospitalizations per 1,000 in 2004, whereas for people 85 years or over there were 47.5 hospitalizations per 1,000 [11]. According to the Medicare Current Beneficiary Survey data, 20.54 percent of Medicare beneficiaries self-reported mental illness or depression in 2003 [12]. Depression has been found to be common among people with other chronic diseases, and its presence can complicate disease management [13]. It is estimated that over 14 million people in the U.S. have been diagnosed with diabetes, a number that increases each year [14]. For the general population with diabetes, direct medical care costs alone were approximately $92 billion in 2002 [14]. Persons with diabetes or cardiovascular disease have a greater prevalence of CKD than persons without either of those conditions [15].

Per capita expenditures increase dramatically with the number of chronic conditions affecting the patient [2,3]. Direct medical care expenditures for people with chronic conditions accounted for approximately 83 percent of U.S. health care dollars in 2001, a per person average which is five times higher than for those without a chronic condition [1]. As the number of chronic conditions increases, the complexity of care and number of different medical providers a patient encounters increases. Use of numerous health care providers can result in redundant and duplicative services (e.g., repeated tests), receipt of conflicting advice, and a lack of overall coordination of care [1]. Not only does the presence of multiple conditions result in higher costs to the Medicare program [3], but the multiplicity of morbidity creates challenges for effectively managing complex medical and supportive care needs. All of these factors contribute to increased costs of care.

The primary objective of this paper is to demonstrate the utility of a new CMS data source, the CCW, for chronic disease research. A secondary objective is to provide a current assessment of the prevalence, utilization, and costs for some of the more common chronic conditions in the Medicare fee for service (FFS) population. This paper explores the burden of multiple chronic conditions in terms of service use and cost to the Medicare program. The care settings commonly used for treating the conditions, as well as the comparative odds of use and average per beneficiary Medicare payments by medical condition, are examined.

## Methods

### CCW Data

CCW administrative claims, enrollment, and chronic condition indicators for 2005 were used in these analyses. Since the CCW data files are already linked by a unique beneficiary key across time and claim type, no beneficiary linkage efforts are required by researchers (e.g., traditionally it has been challenging to link all data for a patient over time because of changes in the Medicare health insurance claim number due to changes in eligibility status). This linkage strategy simplifies examination of the full continuum of care as well as longitudinal studies. Minimal merging of files is required prior to development of the analytic code to address the study objectives.

The CCW contains all Medicare FFS institutional and non-institutional claims, assessment data, and enrollment/eligibility information from January 1, 2000 forward. A random 5% sample of Medicare beneficiaries is the standard data file available to researchers, although the database contains information for 100% of beneficiaries and can be used to select a wide range of cohorts. There are predefined chronic condition indicator variables which are made available to researchers for cohort selection and data extraction, as well as for chronic disease research.

The twenty-one predefined condition indicator variables are coded within the CCW and disseminated to researchers as variables in the Chronic Condition Summary File. Algorithms involving Medicare claims-based utilization information are used to make the chronic condition determinations (i.e., an indicator that the beneficiary received services or treatment for the condition of interest within the specified time period). The identification of each of these conditions is limited to the information available from Medicare administrative claims (e.g., based on ICD-9-CM [16] and HCPCS codes [17]). Treatment information is not available for those enrolled in Medicare managed care plans.

### Study Cohort

Institutional (i.e., inpatient, outpatient, skilled nursing facility, home health, and hospice) and non-institutional (i.e., physician/supplier and durable medical equipment) FFS claims for services provided in 2005 were used in the analyses. The 5% random sample of the Medicare population, based on the standard sampling methodology used by CMS [18], formed the sampling frame for this study, from which a narrower cohort was identified.

The Medicare beneficiary enrollment and eligibility information was obtained from the CCW Beneficiary Summary File, which also contains beneficiary demographic and Medicare coverage information. The predefined chronic condition indicator variables were obtained from the CCW Chronic Condition Summary File. Since these condition indicators are defined using only FFS claims-based criteria (e.g., ICD-9-CM codes, specific combinations of claim types, etc.) and no managed care utilization information, only FFS beneficiaries with Part A and B coverage were included in the cohort. Beneficiaries who were alive on January 1, 2005 and enrolled in Medicare Parts A and B for at least 11 of the 12 months in the year, or until the time of death (i.e., covered for every alive and eligible month, or covered for all except one of the alive and eligible months), and who had one month or less of managed care coverage, were considered eligible for the study cohort. Since this cohort was selected from the random 5% sample, some of whom had the chronic conditions of interest, the findings may be generalized to the larger Medicare FFS population.

### Measures

Nine of the 21 predefined chronic condition indicator variables were used in this study. Four types of cancer were combined into one variable, including female breast, colorectal, prostate, and lung cancer, due to similarities in the patterns of care (e.g., settings used), the desire not to unduly inflate the numbers of distinct disease types being treated simultaneously for a beneficiary, and for simplicity in the analyses. This resulted in six chronic condition variables which were used for these analyses. The diseases represented included cancer, CKD, COPD, depression, diabetes, and HF. A summary of the types of services used to define these conditions is provided in Additional file 1.

The comparison group used throughout this study consisted of the remainder of the random 5% sample who were not receiving treatment for any of these *six *conditions during 2005. Please note that it is possible that some of the beneficiaries within this comparison group may have been receiving treatment for other types of medical conditions (or for any of the other 12 CCW conditions), which were not a part of the current study (i.e., it is not necessarily a disease-free group). The administrative claims data for the study cohort were extracted from the CCW and aggregated by beneficiary using the unique beneficiary identifiers created in the CCW. The resulting beneficiary-level, aggregate claims utilization and cost file was used for all further analyses.

Cancer, COPD, and depression are CCW algorithms which consider services occurring during a one-year look-back period. The CCW uses a two-year look-back period for CKD, diabetes, and heart failure. The algorithms use these look-back periods as the length of time during which a certain service(s) can be provided to a beneficiary for inclusion in the chronic condition category.

Medicare utilization was assessed using each of the claim types. These included inpatient, skilled nursing facility, home health, outpatient, hospice, physician/supplier and durable medical equipment claims. Unique inpatient and skilled nursing facility (SNF) stays were defined as those with a paid Medicare amount and discharge date in 2005, regardless of the reason for the stay. The number of days was calculated by taking the sum of all covered Medicare FFS days of care chargeable to Medicare in 2005. The number of visits (i.e., home health, institutional outpatient, and physician office) was defined as the average number of FFS visits per beneficiary in 2005. Home health (HH) visits were counted using a total visit count variable on the claims. Institutional outpatient (OP) visits were averaged from the sum of the number of outpatient claims. Physician office visits represent the number of evaluation and management visits where the HCPCS ranged from 99201-99205 or 99211-99215, as indicated on the Carrier (physician office) claims.

Costs were defined as total Medicare payment (per claim type), or the sum of all FFS claim payment amounts, per beneficiary for 2005. For each beneficiary, total Medicare payments were summed across all claim types for all services provided during the year, regardless of the diagnosis on the claim. The average Medicare payments per beneficiary were calculated. These population totals and averages were examined for each claim type, then for each of the selected conditions and for beneficiaries with varying numbers of conditions.

### Data Analysis

There are various methods by which the chronic condition indicator variables may be used in the calculation of population prevalence rates for chronic conditions. A technical paper describing some of the basic methods for performing analyses with these indicator variables is available on the CCW web site . The methods used for this study to ascertain prevalence for the chronic conditions, including the rationale for allowing a one month break in FFS Medicare coverage for the study cohort, are more fully described and justified in the technical paper [19]. To summarize, allowing for a one month break in Medicare A or B coverage (or allowing one month of managed care coverage), rather than requiring full Medicare coverage for a 12 month surveillance period, allows for retention of a fair number of beneficiaries in the cohort for whom there is evidence that treatment for the condition(s) of interest occurred. Eleven months (rather than 12 months) FFS coverage may be sufficient for denominator criteria (note that numerator criteria may use different look-back periods) for the purposes of examining population period prevalence of chronic conditions.

The utilization data presented in this paper focus on beneficiary averages rather than simply raw utilization statistics for this cohort. This per capita comparison controls for the number of persons in each category.

For further comparison of utilization across conditions, odds ratios (ORs) were calculated for each care setting. ORs allow for the comparison of the likelihood of the type of care for beneficiaries with a condition, compared to beneficiaries with no condition (i.e., none of the six conditions of interest in this study). For example, the OR for beneficiaries with diabetes receiving inpatient care was computed by dividing the odds of those beneficiaries having an inpatient stay, by the odds of beneficiaries with none of the six conditions having an inpatient stay during the year. The identification of this reference group allows for comparisons regarding the relative importance of the six conditions, and accounts for the fact that the six conditions are not mutually exclusive categories (e.g., beneficiaries may have CKD and diabetes). ORs were also calculated for the comparison of utilization likelihood for beneficiaries with *multiple *conditions to beneficiaries with *none *of the six conditions. Comparisons of utilization across conditions are presented for the most frequently used settings of care.

Cost comparisons of total Medicare payments and *average-per-beneficiary *Medicare payments, by condition and number of conditions present, were also explored in order to more adequately understand the costs of care for beneficiaries with each condition(s). Ratios of means (ROM) were calculated to further compare the differences in average payment amounts per beneficiary by chronic condition and care setting. Each ratio of means was calculated by dividing the average payment amounts per beneficiary for those with the condition, by the average payment amounts per beneficiary for those with none of the six conditions.

## Results

### Demographic Characteristics of Study Population

Table 1 describes the demographic characteristics of the random 5% sample of the Medicare population for 2005, compared to the characteristics of the more restricted, FFS study cohort used in this study. Although the study cohort included only those FFS beneficiaries with 11 of 12 months (or until time of death) of Parts A and B coverage, and minimal managed care coverage (in order to allow for beneficiaries making minor changes in coverage throughout the year), the cohort represents 73.9% of the entire random 5% sample. The beneficiaries in the 5% sample who were excluded from the study cohort were excluded primarily due to having more than one month of managed care coverage, or fewer than 11 months of Part A and B coverage. The demographics, as seen in Table 1, closely mirror those of the random 5% sample.

**Table 1:** Demographic Characteristics of the 2005 Medicare Random 5% Sample and FFS Study Cohort

**Beneficiary Demographics**	**Random 5% Sample^1^**		**Study Cohort^2^**	
	**Number**	**%**	**Number**	**%**

**All**	2,232,528	100.0	1,649,574	100.0
				
**Sex**				
Male	985,629	44.1	71,5925	43.4
Female	1,246,899	55.9	933,649	56.6
				
**Race**				
White	1,870,224	83.8	1,407,709	85.3
Black	220,950	9.9	158,517	9.6
Hispanic	53,325	2.4	32,945	2.0
Asian	37,313	1.7	21,632	1.3
Native American	9,209	0.4	7,083	0.4
Other/Unknown	41,507	1.9	21,688	1.3
				
**Age^3^**				
<65	349,167	15.6	254,457	15.4
65-74	936,988	42.0	641,699	38.9
75-84	670,917	30.1	531,282	32.2
85+	275,456	12.3	222,136	13.5

^1^Includes random 5% sample of Medicare beneficiaries who were eligible for or enrolled in Medicare on or after January 1, 2005.

^2^Includes beneficiaries with at least 11 months of Part A and B coverage and no more than one month of managed care coverage.

^3^Age is calculated based on the age of the beneficiary as of December 31, 2005. If the beneficiary expired, the age is calculated based on age at the time of death.

There are very slight differences in racial composition of the random 5% sample and the study cohort. Younger Medicare beneficiaries (e.g., 65-74 years of age) are somewhat underrepresented in the study cohort. Forty-two percent (42%) of the random 5% sample fall into this age category, compared to 38.9% of the FFS study cohort. This may be partially attributable to the absence of recent accretes into the Medicare program (i.e., for cohort inclusion beneficiaries were required to have had FFS coverage for 11 out of 12 months of the calendar year [or until time of death], therefore, newly eligible beneficiaries with fewer than 11 months of coverage were not included).

### Prevalence of Chronic Conditions and Patterns of Utilization

The prevalence of select chronic conditions for the Medicare FFS study cohort was examined. Table 2 displays the prevalence of the six chronic conditions selected for analysis in this study, along with the annual per beneficiary utilization by condition. These averages include the total number of discharges, days, or visits in 2005, regardless of the diagnosis on the claim(s).

**Table 2:** Condition Prevalence and Per Capita Utilization for 2005, by Condition and Number of Chronic Conditions

**Chronic Condition**	**Prevalence** **(%)**	**Number of Beneficiaries**	**Avg # Inpatient Discharges**	**Avg # Inpatient Days**	**Avg # SNF Days**	**Avg # HH Visits**	**Avg # OP Visits**	**Avg # Physician Office Visits^1^**
Study Cohort^2^		1,649,574	0.39	2.32	1.91	2.77	3.96	6.88
								

**Condition**								

Cancer	6.3%	103,850	0.69	4.29	2.71	3.79	6.39	11.28
CKD	9.0%	149,220	1.35	9.51	7.30	8.84	8.09	10.28
COPD	10.9%	179,554	1.25	8.18	6.29	7.90	6.45	10.24
Depression	11.5%	190,282	0.97	6.49	6.94	6.62	6.81	8.99
Diabetes	24.3%	400,268	0.66	4.18	3.40	5.58	5.53	9.06
HF	17.7%	292,776	1.10	7.28	6.44	8.43	6.64	9.74
								

**# Conditions**								

None	50.7%	836,428	0.12	0.50	0.36	0.77	2.40	4.86
One	29.0%	478,449	0.35	1.80	1.45	2.36	4.40	7.89
Two	12.7%	209,360	0.78	4.65	3.98	5.74	6.30	9.97
Three +	7.6%	125,337	1.76	12.50	10.54	12.70	8.78	11.36

^1^Office visits are identified with Berenson-Eggers Type of Service codes in the line item trailer group(s) in the following ranges of HCPCS (CPT-4) codes: M1A: 99201-99205, M1B: 99211-99215.

^2^Includes random 5% sample of Medicare beneficiaries who were eligible for or enrolled in Medicare on or after January 1, 2005, with at least 11 months of Part A and B coverage and no more than one month of managed care coverage.

The prevalence of the chronic conditions studied is quite high, and variable by condition. The highest prevalence is observed for diabetes with nearly one-fourth of the Medicare FFS study cohort receiving treatment for this condition (24.3 percent). Nearly 18 percent of beneficiaries are receiving care for HF, 11.5 percent for depression, 11 percent for COPD, 9 percent for CKD and 6.3 percent for cancer.

About half of Medicare FFS beneficiaries studied have none of the six chronic conditions (50.7 percent). Twenty-nine percent of beneficiaries are receiving care for only one of these six chronic conditions, 12.7 percent are receiving care for two of the conditions, and 7.6 percent are receiving care for three or more of the conditions.

Beneficiaries with CKD or COPD have the highest yearly per capita number of inpatient stays (see Table 2). Examining inpatient care in a slightly different way, the annual number of inpatient days during 2005 is highest for these two conditions (9.51 and 8.18 days, respectively). The average number of Medicare-covered skilled nursing (SNF) days is highest for those with CKD, followed by those with depression. The largest average number of HH visits is for beneficiaries with CKD, followed by HF. While the largest number of OP visits is for beneficiaries with CKD, the largest average number of physician office visits occurs for people with cancer, followed by CKD and COPD.

Utilization within each care setting soars as the number of chronic conditions increases. The presence of even a single chronic condition escalates the use of services in every setting. For example, the average number of inpatient days per capita in 2005 is 0.5 day for Medicare FFS beneficiaries with none of the six chronic conditions, and 1.8 days for those with one of the conditions. The number of days rises to an average of 12.5 days per year for those with three or more of the six selected chronic conditions. These pronounced differences in utilization are similarly apparent in the home health setting and for physician office visits.

In Table 2 we see that, in some cases, utilization for beneficiaries with *one *of the six listed conditions is higher than utilization for beneficiaries with categorization of *two *conditions, depending on the condition (e.g., the average number of inpatient days for beneficiaries with CKD or COPD is higher than the average number of inpatient days for beneficiaries with two of the six chronic conditions). In order to determine whether it was typical for people with certain chronic conditions to suffer from multiple diseases, prevalence was examined in a slightly different way.

**Figure 1:**
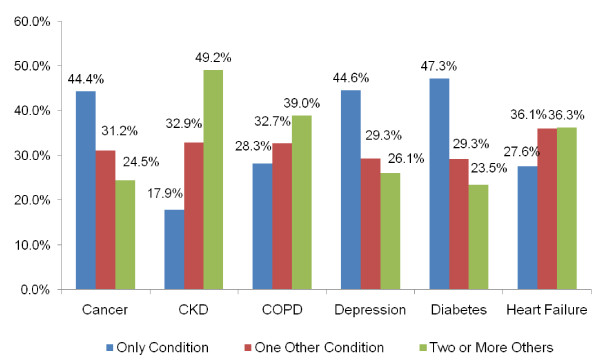
**Proportion of Beneficiaries with Multiple Chronic Conditions**.

Figure 1 illustrates the proportion of beneficiaries with each condition who have only the specified disease, compared to the proportion with one or more of the other six conditions.

It is common to see the presence of multiple chronic conditions with each of the six conditions studied (Figure 1). The highest proportion of beneficiaries with multiple chronic conditions is observed for CKD. Almost 33 percent of beneficiaries with CKD have one of the other conditions, and nearly 50 percent have two or more other chronic conditions. The most common co-occurring conditions were HF (52.9% of those with CKD) and diabetes (51% of those with CKD; data not shown). For diabetes, depression, and cancer, however, beneficiaries are more often diagnosed with only that condition (e.g., for diabetes, 47.3 percent had only diabetes).

### Likelihood of Medical Care Utilization

**Figure 2:**
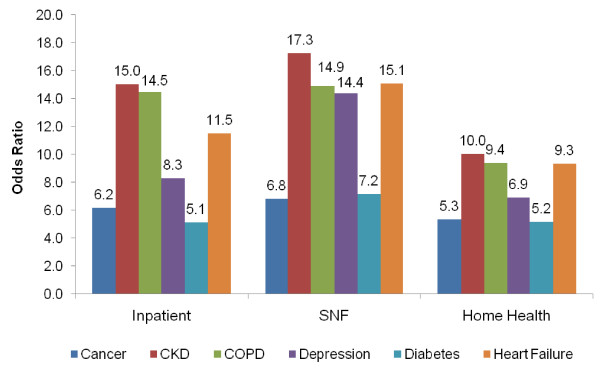
**Likelihood of Utilization (Odds Ratio) by Setting of Care and Chronic Condition**.

The likelihood of receiving particular types of services for beneficiaries with each of the conditions of interest was examined, and compared to the likelihood of utilization for beneficiaries with none of the six chronic conditions. That is, for each condition, the likelihood of utilization (i.e., having an inpatient or SNF visit or HH episode) was compared to the reference group with none of the six conditions. Results are shown in Figure 2.

Medicare beneficiaries with CKD and COPD are much more likely to have an inpatient stay during the year than those without any of these chronic conditions (15 times and 14.5 times more likely, respectively). Those with CKD are 17.3 times more likely to have a Medicare-covered SNF stay, followed by beneficiaries with HF (15.1 times more likely). Beneficiaries with any of the six chronic conditions have a greater likelihood of receiving HH services compared to those without a chronic condition. Among those with chronic conditions, beneficiaries with diabetes and cancer have the lowest likelihood of a HH episode, whereas those with CKD have the highest likelihood of receiving HH visits.

**Figure 3:**
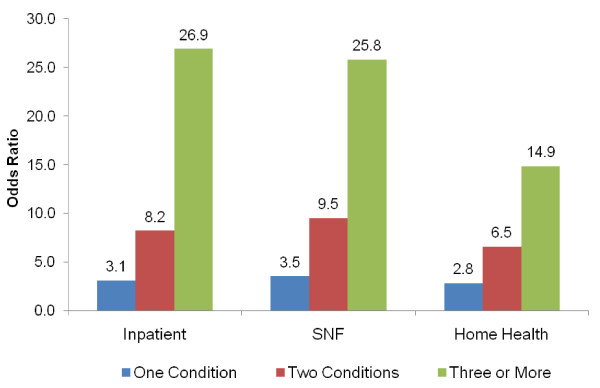
**Utilization Comparison (Odds Ratios) by Setting of Care and Number of Selected Conditions in 2005**.

While Figure 2 allowed for comparison of utilization for beneficiaries with *specific *conditions, Figure 3 displays the comparison of utilization for beneficiaries with *multiple *conditions. Figure 3 demonstrates that beneficiaries with any one of the six conditions are 3.1 times more likely to have an inpatient stay (compared to beneficiaries with no condition), and beneficiaries with three or more conditions are 26.9 times more likely to have an inpatient stay. Similar results are demonstrated for SNF stays. For HH visits, the magnitude of utilization differences for those with multiple conditions is somewhat less pronounced, but still dramatic. Beneficiaries with one condition are 2.8 times more likely, and those with three or more conditions are 14.9 times more likely, to have a HH visit than beneficiaries with none of the conditions.

### Medicare Payments for Beneficiaries with Chronic Conditions

Higher utilization of services is generally associated with higher costs. Nonetheless, it is helpful to examine overall Medicare payment amounts for treating beneficiaries with each of the chronic conditions, as well as the average per beneficiary costs to Medicare associated with each of the claim types. Table 3 details the total FFS Medicare payment amounts by condition.

**Table 3:** Total and Average per Beneficiary Medicare Payments in 2005 by Claim Type for Selected Conditions

	**Total Medicare Payments** **(round to millions)**	**Average Payment** **per Beneficiary**
**Total^1^**	$12,989	$7,874

		
**Claim Type**		

Inpatient	$5,513	$3,342
SNF	$911	$552
Hospice	$321	$194
HH	$615	$373
OP	$1,605	$973
Physician/supplier	$3,588	$2,175
Durable medical equipment	$437	$265
		
**Type of Condition^2^**		

Cancer	$1,668	$16,057
CKD	$3,980	$26,671
COPD	$3,844	$21,409
Depression	$3,210	$16,869
Diabetes	$5,236	$13,082
HF	$6,015	$20,545
		
**# Conditions**		

None	$2,359	$2,820
One	$3,431	$7,172
Two	$3,126	$14,931
Three or More	$4,073	$32,498

^1^Represents total Medicare payment for all claims regardless of the diagnosis on the claim. Includes beneficiaries in study cohort with at least 11 months of Part A and B coverage and no more than one month of managed care coverage.

^2^Beneficiaries may be counted in more than one chronic condition category.

The highest Medicare payment amounts for the study cohort are derived from inpatient stays, followed by physician/supplier services. The lowest is for hospice care. The average per beneficiary Medicare payments are highest for beneficiaries with CKD ($26,671 in 2005). Payments are also high for those with COPD ($21,409) and HF ($20,545). This is in stark comparison to an average per beneficiary payments for those without any of the six chronic conditions ($2,820 per year).

As the number of chronic conditions increases, the average per beneficiary Medicare payment amounts increase dramatically (Table 3). The annual Medicare payment amounts for a beneficiary with only one of the chronic conditions is $7,172. For those with two conditions, payment jumps to $14,931, and for those with three or more conditions, the annual Medicare payments per beneficiary is $32,498.

Comparing the prevalence data from Table 2 to the average per beneficiary payment data from Table 3, it is apparent that a disproportionate share of Medicare payments is spent treating beneficiaries with chronic conditions. Beneficiaries with three or more chronic conditions account for merely 7.6 percent of the Medicare FFS population, yet they account for 31 percent of total Medicare payments of the study cohort (calculated by dividing $4,073,000,000 for 3+ conditions by $12,989,000,000 for the study cohort, see Table 3).

Some conditions may result in higher Medicare payments than others. For each claim type, we can determine how much more costly it is to care for beneficiaries with select conditions or multiple conditions, compared to beneficiaries with none of the conditions. This is accomplished by calculating a ratio of means (ROM) to quantify the magnitude of the average payment differences for treating beneficiaries with different types and numbers of chronic conditions. Results are displayed in Table 4.

**Table 4:** Relative Medicare Payments^1 ^in 2005 by Claim Type for Selected Chronic Conditions

**Condition^2^**	**Inpatient**	**SNF**	**HH**	**Hospice**	**OP**	**Physician/** **Supplier**	**DME**
Cancer	7.4	7.2	4.5	5.1	5.2	4.7	4.8
CKD	15.4	18.5	9.8	4.8	8.9	4.6	9.6
COPD	12.8	16.0	8.7	4.4	4.2	3.9	13.0
Depression	9.1	17.5	7.5	3.6	4.1	3.2	6.4
Diabetes	6.9	8.6	6.0	2.5	3.8	2.8	7.4
HF	11.9	16.5	9.3	5.5	4.9	3.6	8.8
							

**# Conditions**							

None	1.0	1.0	1.0	1.0	1.0	1.0	1.0
One	3.1	3.8	2.9	2.2	2.3	2.0	3.7
Two	7.7	10.3	6.5	4.0	4.3	3.2	7.7
Three +	19.4	26.6	13.7	6.1	8.0	5.5	14.0

^1^Relative payments are calculated using a ratio of means (ROM): average payments for beneficiaries with the condition divided by the average payments for beneficiaries with none of the selected conditions.

^2^Beneficiaries may be counted in more than one chronic condition category and/or claim type.

The average Medicare payment amount for inpatient care is 15.4 times higher for someone with CKD than for beneficiaries who had none of the conditions. The payments for SNF care are highest for those with CKD, followed by those with depression, and HF, compared to those with none of these chronic conditions. The highest per beneficiary Medicare payments for HH services are observed for those with CKD and HF. Beneficiaries with HF and cancer have the highest per capita Medicare payments for hospice care, compared to those with none of the conditions. OP care is 8.9 times more costly per beneficiary for those with CKD compared to beneficiaries with none of the conditions. Total Medicare payments for physician/supplier services are highest for beneficiaries with cancer and CKD.

The high cost of caring for beneficiaries with CKD may be due, in part, to the high prevalence of end stage renal disease (ESRD) in this population. Among those with CKD, 10.8 percent also have ESRD (data not shown), and this subpopulation accounts for 22.7 percent of the Medicare costs for those with CKD (regardless of diagnosis on claim). The average per beneficiary cost in 2005 for those with CKD and without ESRD is $23,135 and $55,780 for those with both CKD and ESRD. The ESRD co-occurrence with CKD is substantially higher than the observed rate for any of the five other chronic conditions, with an ESRD prevalence ranging from 3.4 percent in the HF cohort to 0.7 percent in the cancer cohort.

For beneficiaries with one or more chronic condition(s), Medicare payments increase dramatically as the number of conditions increases. This relationship is similar for all claim types. For more acute settings of care (e.g., inpatient, SNF, HH), average per beneficiary payment amounts grow exponentially as the number of chronic condition(s) increases. For physician/supplier services or hospice care, average payments increase in a more linear way as the number of chronic conditions increase.

## Discussion

As expected, based on earlier findings in the literature, prevalence of the six chronic conditions included in this study is quite high in the Medicare FFS population. Almost fifty percent of beneficiaries have at least one of the six chronic conditions considered in this study. Nearly one-fourth of the Medicare FFS population is receiving treatment for diabetes.

In addition, the prevalence of multiple chronic conditions is significant. For CKD, it is common for beneficiaries to have multiple chronic conditions, with nearly half of these beneficiaries suffering from two or more other chronic conditions. For those with CKD, we also observe a high level of service use and high cost to Medicare per beneficiary.

For the Medicare FFS cohort studied, the inpatient care setting accounts for the largest proportion of Medicare spending. CKD is the condition with the highest average per beneficiary Medicare payments at $26,671 in 2005. This high cost is at least partially attributable to the high prevalence of ESRD within the CKD cohort. Beneficiaries with three or more chronic conditions have average Medicare payments of $32,498.

This study was conducted using a Medicare FFS population. Administrative data were used to infer disease status. FFS claims were analyzed to determine whether there was an indication of receiving evaluation of or treatment for the condition of interest. There is always a risk with administrative data sources that a beneficiary may be erroneously classified as not having one of these conditions due to lack of treatment for the condition (e.g., inability to obtain care or presence of subclinical disease). The CCW does not contain managed care claims (or encounter data), therefore it was not possible to ascertain whether the prevalence of chronic conditions illustrated in this study of a Medicare FFS population is similar to the prevalence in the Medicare managed care population.

## Conclusion

The CCW data files have tremendous value for ongoing evaluation of disease management programs and initiatives. The longitudinal data and beneficiary linkage within the CCW are features of this data source which make it ideal for further studies regarding disease prevalence and progression over time. As additional years of administrative data are accumulated in the CCW, the expanded history of beneficiary services increases the value of this already rich data source. While the findings in these data presentations support the types of conditions and care settings typically addressed by comprehensive chronic disease management programs, the findings also demonstrate a need for further exploration of utilization, costs, and outcomes for certain conditions.

## Abbreviations

**CCW**: Chronic Condition Data Warehouse; **CKD**: Chronic kidney disease; **CMS**: Centers for Medicare and Medicaid Services. Administers U.S. Medicare Program. Part of the U.S. Department of Health and Human Services; **COPD**: Chronic obstructive pulmonary disease; **CPT-4**: Current Procedural Terminology^®^. Version 4. is a uniform coding system consisting of descriptive terms and identifying codes that are used primarily to identify medical services and procedures furnished by physicians and other health care professionals. CPT^® ^is a registered trademark of the American Medical Association.; **DME**: Durable Medical Equipment; **DX**: Diagnosis; **FFS**: Fee-for-service; **HCPCS**: Healthcare Common Procedure Coding System (HCPCS) Level I of the HCPCS is comprised of Current Procedural Terminology (CPT-4), a numeric coding system maintained by the American Medical Association (AMA).; **HF**: Heart failure; **HH**: Home health care; **ICD-9-CM**: International Classification of Diseases, Ninth Revision, Clinical Modification (ICD-9-CM) is based on the World Health Organization's Ninth Revision, International Classification of Diseases (ICD-9).; **OR**: Odds ratio; **OP**: Outpatient (hospital facility); **ROM**: Ratio of means; **SNF**: Skilled nursing facility

## Competing interests

The authors declare that they have no competing interests.

## Authors' contributions

KMS conceived of the study, participated in the design and drafting of all sections of this manuscript, and assisted with literature review and data verification. BEO provided significant contribution to the design of the study, performed all statistical analyses, edited all tables and figures, as well as the manuscript. DD participated in the design of the study, the literature review, preparing all data tables and figures, and editing all portions of this manuscript.

## Supplementary Material

Additional file 1 **Definitions of Chronic Conditions used in Analyses.** Click here for file
