# Supplementary material for: Prevalence of multiple chronic conditions in the United States' Medicare population
Source: Health Qual Life Outcomes. 2009 Sep 8;7:82. doi: 10.1186/1477-7525-7-82 (PMC2748070; doi:10.1186/1477-7525-7-82)
Supplement: Additional file 1 — Definitions of Chronic Conditions used in Analyses. [file 1477-7525-7-82-S1.doc]

| **Condition** | **Reference Time Period** | **Valid ICD-9/CPT4/HCPCS Codes** | **Number/Type of Claims to Qualify** |
| --- | --- | --- | --- |
| Chronic Kidney Disease (CKD) | 2 years | DX 016.00, 016.01, 016.02, 016.03, 016.04, 016.05, 016.06, 095.4, 189.0, 189.9, 223.0, 236.91, 250.40, 250.41, 250.42, 250.43, 271.4, 274.1, 283.11, 403.01, 403.11, 403.91, 404.02, 404.03, 404.12, 404.13, 404.92, 404.93, 440.1, 442.1, 572.4, 580.0, 580.4, 580.81, 580.89, 580.9, 581.0, 581.1, 581.2, 581.3, 581.81, 581.89, 581.9, 582.0, 582.1, 582.2, 582.4, 582.81, 582.89, 582.9, 583.0, 583.1, 583.2, 583.4, 583.6, 583.7, 583.81, 583.89, 583.9, 584.5, 584.6, 584.7, 584.8, 584.9, 585, 585.1, 585.2, 585.3, 585.4, 585.5, 585.6, 585.9, 586, 587, 588.0, 588.1, 588.81, 588.89, 588.9, 591, 753.12, 753.13, 753.14, 753.15, 753.16, 753.17, 753.19, 753.20, 753.21, 753.22, 753.23, 753.29, 794.4 (any DX on the claim) | At least 1 inpatient, SNF or HH claim or 2 OP or Carrier claims with DX codes during the 2-yr period |
| Chronic Obstructive Pulmonary Disease (COPD) | 1 year | DX 491.0, 491.1, 491.20, 491.21, 491.22, 491.8, 491.9, 492.0, 492.8, 494.0, 494.1, 496 (any DX on the claim) | At least 1 inpatient, SNF, HH or 2 OP or Carrier claims with DX codes during the 1-yr period |
| Depression | 1 year | DX 296.20, 296.21, 296.22, 296.23, 296.24, 296.25, 296.26, 296.30, 296.31, 296.32, 296.33, 296.34, 296.35, 296.36, 296.50, 296.51, 296.52, 296.53, 296.54, 296.55, 296.56, 296.60, 296.61, 296.62, 296.63, 296.64, 296.65, 296.66, 296.89, 298.0, 300.4, 309.1, 311 (any DX on the claim) | At least 1 inpatient, SNF, HH, OP or Carrier claim with DX codes during the 1-yr period |
| Diabetes | 2 years | DX 250.00, 250.01, 250.02, 250.03, 250.10, 250.11, 250.12, 250.13, 250.20, 250.21, 250.22, 250.23, 250.30, 250.31, 250.32, 250.33, 250.40, 250.41, 250.42, 250.43, 250.50, 250.51, 250.52, 250.53, 250.60, 250.61, 250.62, 250.63, 250.70, 250.71, 250.72, 250.73, 250.80, 250.81, 250.82, 250.83, 250.90, 250.91, 250.92, 250.93, 357.2, 362.01, 362.02, 366.41 (any DX on the claim) | At least 1 inpatient, SNF or HH claim or 2 OP or Carrier claims with DX codes during the 2-yr period |
| Heart Failure | 2 years | DX 398.91, 402.01, 402.11, 402.91, 404.01, 404.11, 404.91, 404.03, 404.13, 404.93, 428.0, 428.1, 428.20, 428.21, 428.22, 428.23, 428.30, 428.31, 428.32, 428.33, 428.40, 428.41, 428.42, 428.43, 428.9 (any DX on the claim) | At least 1 inpatient, OP or Carrier claim with DX codes during the 2-yr period |
| *The following types of cancer were combined for the purposes of these analyses, and are herein referred to as Cancer.* | | | |
| Female Breast Cancer | 1 year | DX 174.0, 174.1, 174.2, 174.3, 174.4, 174.5, 174.6, 174.8, 174.9, 233.0 (any DX on the claim) | At least 1 inpatient or 2 OP or Carrier claims with DX codes during the 1-year time period |
| Colorectal Cancer | 1 year | DX 154.0, 154.1, 153.0, 153.1, 153.2, 153.3, 153.4, 153.5, 153.6, 153.7, 153.8, 153.9, 230.3, 230.4 (any DX on the claim) | At least 1 inpatient or 2 OP or Carrier claims with DX codes during the 1-year time period |
| Prostate Cancer | 1 year | DX 185, 233.4 (any DX on the claim) | At least 1 inpatient or 2 OP or Carrier claims with DX codes during the 1-year time period |
| Lung Cancer | 1 year | DX 162.0, 162.2, 162.3, 162.4, 162.5, 162.8, 162.9, 231.2 (any DX on the claim) | At least 1 inpatient or 2 OP or Carrier claims with DX codes during the 1-year time period |
